# Vaccination coverage for measles, mumps, and rubella in a tertiary hospital of Porto Alegre, state of Rio Grande do Sul

**DOI:** 10.47626/1679-4435-2020-510

**Published:** 2021-02-11

**Authors:** Diêgo da Silva Dantas, Damásio Macedo Trindade, Maria Carlota Borba Brum

**Affiliations:** 1 Programa de Residência Médica em Medicina do Trabalho, Hospital de Clínicas de Porto Alegre (HCPA), Porto Alegre, RS, Brazil; 2 Faculdade de Medicina, Universidade Federal do Rio Grande do Sul, Porto Alegre, RS, Brazil; 3 Serviço de Medicina Ocupacional, HCPA, Porto Alegre, RS, Brazil

**Keywords:** measles, mumps, rubella, vaccination, professionals

## Abstract

**Introduction:**

Measles, mumps, and rubella are viral contagious diseases preventable by the MMR vaccine. MMR is a part of the immunization schedule of the Brazilian Ministry of Health, which recommends 2 doses of the vaccine for professionals working in health care facilities.

**Objectives:**

To determine the vaccination coverage for measles, mumps, and rubella among active professionals of a tertiary hospital in the city of Porto Alegre, state of Rio Grande do Sul; to analyze the importance of vaccinating professionals that work at health care facilities; to detect and highlight high-risk services for contagious diseases according to patient profiles - children, pregnant and puerperal women, immunocompromised individuals.

**Methods:**

This is a descriptive transversal study that analyzed the database of the Occupational Medicine Service of Hospital de Clínicas de Porto Alegre considering active professionals in January 2019.

**Results:**

We evaluated the vaccination records of 7802 active professionals of this hospital; 52% of them had taken at least 1 dose of the MMR vaccine (1 or 2 doses) and 47% had not been vaccinated. Among high-risk services, 56.4% of the professionals had had at least 1 MMR dose, while 43.4% had not been vaccinated.

**Conclusions:**

This study revealed that the vaccination coverage for measles, mumps, and rubella at the analyzed health care facility was still far from the ideal; however, the Occupational Medicine Service has been making efforts to gradually broaden this coverage.

## INTRODUCTION

Measles, mumps, and rubella are highly contagious viral diseases transmitted by respiratory secretions produced by sneezing, coughing, talking, or breathing, and that are more easily propagated in closed or crowded spaces^1^. All three diseases are considered preventable through vaccination.

Measles is an acute exanthematic disease that can present complications and lead to death, particularly in children that are undernourished or under 1 year old. Contagion happens from person to person through respiratory secretions, in a period of 4 to 6 days before and up to 4 days after the appearance of the exanthem^2^. In Brazil, the last measles outbreaks had been registered in 2015 in the states of Ceará, São Paulo, and Roraima, and in 2016 the country received a certification of regional measles elimination by the World Health Organization (WHO). However, Venezuela has been facing a measles outbreak since July 2017, and due to intense migratory movement, the virus has reached Brazil once again. In 2018, approximately 10 000 measles cases were confirmed in Brazil, of which 45 happened in the state of Rio Grande do Sul^2^.

Mumps (infectious parotitis) can be transmitted through the saliva or its droplets; it is rarely spread through objects contaminated with nasal or mouth secretions. This disease is transmissible 1 week before symptoms and up to 10 days after the onset of the clinical presentation. Its main characteristic is the swelling of salivary glands, especially the parotid salivary gland, followed by pain when eating or drinking acidic liquids, fever, headache, muscle pain, and loss of appetite^3^.

In Brazil, Mumps is not a mandatory notifiable disease, which hampers the evaluation of its national prevalence. Nonetheless, since states and cities can report important regional health problems, the state of Rio Grande do Sul has included mumps as a state-level issue. In this state, there was a high incidence of mumps in the 1980s, with 2- to 4-year cycles. At the end of the 1990s and throughout the 2000s, the incidence of this disease notably decreased, which probably stemmed from the establishment of the MMR vaccine in the private service in 1997. Since 2015, the incidence of mumps has increased and its occurrence has changed: before the vaccine, it struck children under 10 years old, whereas now it reaches 15- to 19-year-old adolescents and 20- to 29-year-old adults^4^.

Rubella is also an exanthematic disease whose higher epidemiological importance results from the fact that it presents risks of abortions, stillbirths, and congenital anomalies such as cardiopathies, cataract, and deafness when contracted by pregnant women. Similarly to mumps and measles, it is transmitted by contact with nasopharyngeal secretions of infected people; indirect transmission through objects contaminated with nasopharyngeal secretions, blood, and urine is rare. The virus is transmissible from 5 to 7 days before until 7 days after the appearance of the exanthem^5^.

In 2015, Brazil received a certification of regional rubella elimination by the WHO. The last autochthonous cases of rubella were detected in 2008 and those of congenital rubella syndrome, in 2009. Since globalization has resulted in the circulation of more people between countries, and consequently of the virus worldwide, health services should remain vigilant in order to properly detect new cases and implement measures for interrupting disease transmission and spread^4^.

The Brazilian Ministry of Health offers the MMR vaccine against measles, mumps, and rubella through the Unified Health System (SUS) in over 36 000 vaccination units throughout the country and according to the National Vaccination Schedule. The vaccine should be administered to children as young as 12 months old, in 2 doses with a minimum interval of 30 days. The Brazilian Immunization Society (SBIm) recommends 2 MMR doses for professionals that work in health care facilities, except when there are contraindications^6^.

The National Association of Occupational Health (ANAMT) recommends universal vaccination from 12 months of age (according to a schedule of 2 doses and a 30-day interval) for the control of all three diseases. A single dose could result in incomplete protection; a third dose can be considered for people in risk groups during mumps outbreaks^1^.

The Brazilian Regulatory Standard 9 (NR 9) presents the Environmental Risk Prevention Program (PPRA) and includes fungi, bacteria, protozoans, and viruses as biological risks. It states that the PPRA should include measures of risk control and methods for their evaluation, but does not approach immunization as a prevention strategy^7^. NR 32 (Health and Safety at Work in Health Services), on the other hand, states that the Occupational Health Control Program (PCMSO) should include a vaccination program in addition to the aspects mentioned by NR 7, in accordance with recommendations by the Ministry of Health^8^.

This study aimed to determine the vaccination coverage for measles, mumps, and rubella among active professionals at a major tertiary hospital in the city of Porto Alegre, focusing on high-risk areas for these contagious diseases according to the patient profiles. We would also like to highlight the importance of the vaccination of professionals that work at health care facilities.

## METHODS

This is a descriptive transversal study performed at the Occupational Medicine Service (SMO) of Hospital de Clínicas de Porto Alegre (HCPA). HCPA is a high-complexity public health institution established in 1971 that is part of the network of university hospitals of the Ministry of Education and is linked to the Federal University of Rio Grande do Sul.

Based on the STARH system, used in the management of the hospital’s Specialized Services in Occupational Safety and Health (SESMT), we retrieved data on all 322 sectors of HCPA regarding MMR vaccination doses registered for each professional, maintaining the confidentiality of names, occupations, and other personal data. Our initial sample comprised all professionals registered at the SMO in January 2019: a total of 8546 people. In addition to active professionals, this initial sample included residents and professors that had vaccination records in the STARH system. After excluding professionals that were on leave in January 2019, the final sample contained 7802 participants.

After evaluating the profiles of patients cared for at the hospital, we defined 5 risk areas based on the following criteria: (1) patients with higher chances of contracting contagious diseases due to inherent conditions (children, immunocompromised cancer or transplant patients); (2) patients with higher chances of contracting contagious diseases due to lack of vaccination (unvaccinated children); (3) pregnant women (who cannot receive the MMR vaccine and present higher risks of serious effects of these contagious diseases).

The study was approved by the research ethics committee of HCPA (Certificate of Presentation for Ethics Consideration [CAAE] No. 16762619.0.0000.5327). Researchers signed a free and informed consent form for exempt research and a commitment to use institutional data.

## RESULTS

**Figure 1:**
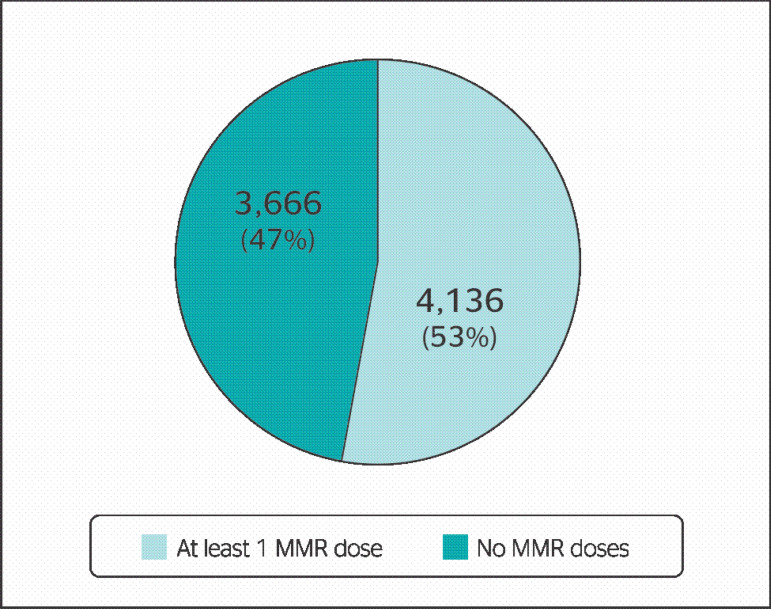
Registers of MMR vaccine doses of all professionals in the analyzed sample.

Among the 7802 professionals included in this study, 1828 (23.4%) had received only 1 MMR dose, 2308 (29.6%) had had both doses, and 3666 (47%) did not have vaccination records for MMR. Considering the professionals that had been vaccinated with 1 or 2 doses, Figure 1 illustrates the numbers of vaccinated and unvaccinated professionals.

**Figure 2:**
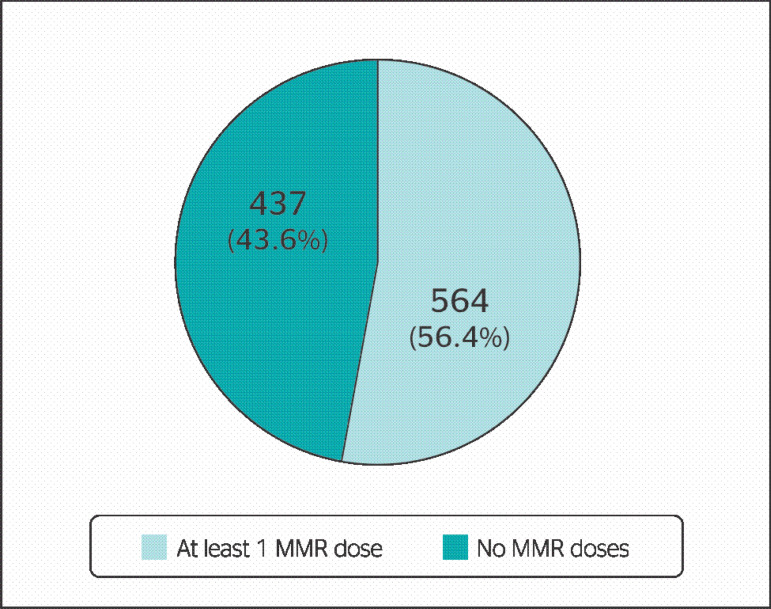
Registers of MMR vaccine doses of professionals assigned in risk areas.

Regarding 1001 professionals assigned to risk areas, 257 (25.7%) had a record of only 1 dose, 307 (30.7%) had received both doses, while 437 (43.6%) did not have a record of MMR vaccination. Figure 2 illustrates that 564 (56.4%) professionals of risk areas had 1 or 2 doses of the vaccine.

In pediatric care, out of 626 professionals, 155 (24.8%) had a record of 1 vaccine dose, 197 (31.4%) had received 2 doses, and 274 (43.8%) did not have a record of MMR vaccination. Professionals with at least 1 dose accounted for 352 (56.2%) of all professionals in this sector.

Considering professionals that worked in obstetric care, 62 (32.6%) out of 190 participants had a record of one dose of the MMR vaccine, while 44 (23.2%) had received both doses and 84 (44.2%) had not been vaccinated. Professionals that had been vaccinated at least once represented 106 (55.8%) of all professionals in this service.

Professionals that worked in cancer and transplant patient care accounted for 139 participants; 27 (19.4%) had been vaccinated with one dose of the MMR vaccine, while 69 (42.4%) had received both doses and 53 (38.1%) had never been vaccinated. We observed that 86 (61.8%) participants had been vaccinated with 1 or 2 doses of the vaccine.

**Figure 3:**
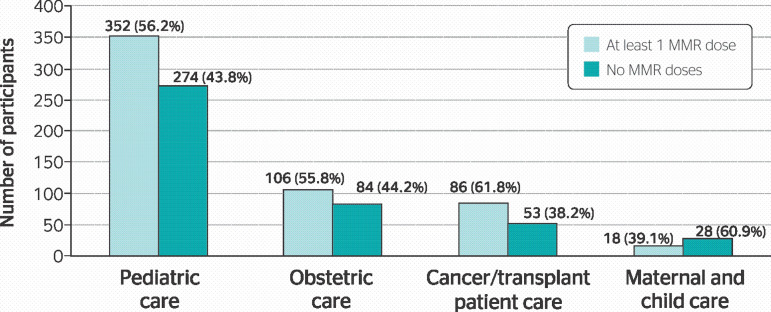
Registers of MMR vaccine doses of professionals assigned in each risk area.

In maternal and child care, out of 46 professionals, 11 (23.9%) had received one MMR dose, 7 had received both doses (15.2%), and 28 (60.9%) had no record of MMR vaccination. Professionals who had received 1 or 2 doses of the vaccine accounted for 18 (39.1%) of the participants in this service. Figure 3 presents data on the vaccination coverage in each of the risk areas of this study.

## DISCUSSION

Studies regarding the MMR vaccination coverage are generally performed with data from pediatric populations. Among health professionals, the most studied immunization aspect is that of hepatitis B, which is verified through vaccination schedules and anti-HBs confirmation tests. Some authors have also studied the immunization profiles of students in health-related undergraduate courses such as nursing and medical schools before their entrance in the job market^9-11^.

Pinto et al.^12^ evaluated the vaccination status of 47 professionals working at Family Health Strategy (ESF) units in a city in the state of Ceará. The authors reported that 55% of the participants of their study had been vaccinated with both MMR doses^12^. In the state of Bahia, another study evaluated 5 cities and 3084 workers at primary and secondary care and verified that 72.8% of the participants had a complete vaccination schedule against measles, mumps, and rubella^13^.

In our study, out of 7802 evaluated participants, almost half (47%) had no record of an MMR vaccine. When bringing together the participants with a record of only one vaccine dose (1828), we observed that the percentage of professionals with an incomplete vaccination schedule against measles, mumps, and rubella reached 40.4%. Among the risk areas defined by this study (pediatric, obstetric, cancer, and transplant patients, in addition to maternal and child care), vaccination coverage was also insufficient. Out of 1001 workers, almost half (43.6) did not have a record of MMR vaccination, and when considering also those who had received only 1 dose of the vaccine, the percentage of participants with incomplete vaccination schedules reached 69.3%.

On the other hand, when grouping together the individuals that had received 1 or 2 doses of the vaccine, the percentage of workers with at least 1 vaccine dose enhances considerably, generating the saying “one dose is better than none.” In fact, according to the WHO and regarding the MMR vaccine, 1 dose administered after 12 months of age presents approximately 94% efficacy for measles, while 2 doses can reach 100%. Serological and epidemiological evidence indicates that the vaccine induces long-term measles immunity in most people: approximately 95% of vaccinated people have detectable antibodies 11 years after the first dose and 15 years after the second dose. The rubella component of this vaccine induces cellular and humoral immunity; approximately 95% of people develop immunity after 1 dose, and this number reaches 99% after 2 doses. Antibodies can be detected in individuals that received 1 dose after more than 16 years, and among those that received 2 doses, up to 100% had detectable antibody levels 12 to 15 years after vaccination^14^.

Immunity against mumps induced by the MMR vaccine stumbles upon different problems. Approximately 94% of the pediatric population develops detectable antibodies after vaccination. Most of those who receive the second dose have a 4-fold increase in antibody titers, and the percentage of people with low or undetectable titers is reduced from 20% to 4% 6 months after vaccination. However, although antibody titers are frequently used when evaluating mumps immunity, there is no available serological test that can be consistently and reliably used in the measurement of mumps immunity. The immune response to mumps is known to be both humoral and cellular, but correlations with protection have not yet been established^14^. Altogether, these data suggest that there is no indication for ordering routine antibody measurements in workers of health care facilities. Therefore, the documental evaluation of mumps immunization is currently only performed using proof of MMR vaccination.

The American Centers for Disease Control and Prevention (CDC) state that the following individuals should not receive the MMR vaccine: those with any major or potentially fatal allergies; those who have had allergies with risk of death after an MMR dose or with any of the components of the vaccine; pregnant women (they should wait until after delivery) or planning to get pregnant (they should postpone pregnancy to at least 1 month after vaccination); immunocompromised people due to diseases (cancer or human immunodeficiency virus/acquired immunodeficiency syndrome [HIV/Aids]) or treatments (radiotherapy, immunotherapy, corticosteroids, or chemotherapy); those that have first-degree relatives with a history of immunodeficiency (individualized care is recommended); individuals with disorders that cause them to bleed easily; and those who received transfusions of blood components in the last 3 months^15^.

The CDC also recommends that verbal accounts of vaccination should not be accepted without documental proof as evidence of immunity. The agency considers that health care professionals should provide records of 2 doses of the MMR vaccine or laboratorial confirmation of their immunity status as evidence. In cases where the individual cannot present these documents as evidence, vaccination is recommended. The MMR vaccine is safe and there is no risk in receiving “extra” doses, according to the CDC^15^.

The SMO of HCPA provides year-round MMR vaccination for all its professionals since 2018. Occupational medicine residents and physicians are required to verify the vaccination status of professionals in all occupational examinations, including those performed in the return to work and change of function. These professionals should also recommend vaccination, which could be performed within the service if there are no contraindications. In addition, vaccination campaigns are performed periodically, aiming to reach as many professionals as possible.

It is of utmost importance to recognize the fact that unvaccinated workers jeopardize not only their health, but also that of the patients around them, especially those that are less resistant to contagious diseases or that have contraindications to vaccination. Vaccine hesitancy, that is, the delay or refusal in accepting recommended vaccines (when they are available in health services), is currently a growing concern worldwide^16^. In the United States, a literature review published in 2016 used data starting from when measles was declared eradicated in the country (January 1, 2000) and reported 1416 cases, of which over half (56.8%) did not have measles vaccination records. This phenomenon of vaccine refusal has been associated to an increase in measles cases^17^.

There are various reasons that could influence a person to refuse vaccination. These aspects are associated to different patterns of vaccination behavior. Some studies suggest that educational measures could decrease refusal in some cases, which could be useful in the specific approach to occupational health^18^.

## CONCLUSIONS

This study revealed that the vaccination coverage against mumps, rubella, and measles in the evaluated institution is still far from ideal, although the SMO has been putting efforts into broadening this coverage. Our study could serve as a basis for subsequent work and subsidize strategies and plans of action for increasing the vaccination coverage among professionals working at HCPA, especially those assigned in risk areas.
